# Interleukin-22 promotes aerobic glycolysis associated with tumor progression via targeting hexokinase-2 in human colon cancer cells

**DOI:** 10.18632/oncotarget.15913

**Published:** 2017-03-06

**Authors:** Yulin Liu, Fan Xiang, Yongming Huang, Liang Shi, Chaojie Hu, Yiming Yang, Di Wang, Nan He, Kaixiong Tao, Ke Wu, Guobin Wang

**Affiliations:** ^1^ Department of Gastrointestinal Surgery, Union Hospital, Tongji Medical College, Huazhong University of Science and Technology, Wuhan 430022, China; ^2^ Department of Clinical Laboratory, Union Hospital, Tongji Medical College, Huazhong University of Science and Technology, Wuhan 430022, China

**Keywords:** interleukin-22, colon cancer, aerobic glycolysis, proliferation, hexokinase-2

## Abstract

Interleukin-22 has been explored extensively in human cancer, but its functions and underlying mechanisms are incompletely understood. Here, we show that aberrant interleukin-22 expression facilitates aerobic glycolysis in colon cancer cells. Elevated interleukin-22 mRNA expression was observed and positively correlated with hexokinase-2 in colon cancer tissues. *In vitro*, interleukin-22 enhanced glucose consumption and lactate production via targeting hexokinase-2 in colon cancer cells. Moreover, the transcriptional factor c-Myc and signal transducer and activator of transcription 3 were involved in interleukin-22-induced up-regulation of hexokinase-2. We further demonstrated that hexokinase-2 partly accounted for interleukin-22-mediated cellular proliferation in DLD-1 cells. *In vivo*, our data demonstrated that interleukin-22 significantly promoted tumor growth along with elevated expression of c-Myc and hexokinase-2 in mice. In summary, our findings provide a new perspective on the pro-inflammatory cytokine interleukin-22 in promoting aerobic glycolysis associated with tumor progression in human colon cancer cells.

## INTRODUCTION

Colorectal cancer (CRC) ranks as the third most common cancer and the fourth leading cause of death in the world [1]. Currently, curative surgical resection is still the optimal treatment for primary CRC. However, the lack of an effective therapy leads to tumor recurrence and metastasis after surgery. Therefore, there is an urgent need to understand molecular mechanisms underlying the development and progression of CRC.

In contrast to normal cells, cancer cells rely on glycolysis as the main source of energy, regardless of oxygen availability, a phenomenon referred to as aerobic glycolysis (the Warburg effect) [2]. Aerobic glycolysis generates less ATP than oxidative phosphorylation, but it can rapidly provide sufficient energy and biosynthetic precursors for cellular proliferation [3–5]. Based on the elevated glucose absorption in tumor tissue, an imaging technique called positron-emission tomography (PET), which uses a glucose analogue tracer (F^18^-fluorodeoxyglucose), has been developed and is widely used in oncological examinations [6]. Although the Warburg effect has been well known for some time, the underlying mechanisms have remained largely elusive. Recent studies suggest that cancer-specific metabolism plays a vital role in proliferation, invasion and chemo-resistance of cancer cells [7–9]. Therefore, deciphering the underlying mechanism by which cancer cells adopt aerobic glycolysis could potentially contribute to understanding the biological characteristic of tumor cells and aid in the development of therapies to treat human malignancies [10].

Recently, the association between inflammatory environment and cancer-specific metabolism has been attracting increased attention. Fibroblast growth factor 21 enhances glucose uptake through the induction of glucose transporter (Glut) 1 expression in adipocytes [11]. Interleukin (IL) 17 and tumor necrosis factor α were reported to stimulate glucose metabolism cooperatively in colorectal cancer cells [12]. Notably, recent studies showed that the pro-inflammatory cytokine IL-6 promoted aerobic glycolysis by activating signal transducer and activator of transcription (STAT) 3 [13, 14]. As a potent STAT3 activator, IL-22 is a member of the IL-10 cytokine family, principally produced by Th22 and Th17 subsets of CD4^+^ T cells which play important roles in tumor development [15–17]. IL-22 signaling is transduced through a heterodimer receptor consisting of IL-22R1 and IL-10R2, leading to activation of downstream signaling pathway, particularly phosphorylation and activation of the transcription factor STAT3 [18, 19]. Interestingly, IL-22R1 expression is restricted to epithelial cells, such as those in the skin, liver, colon and kidney [20]. Numerous reports have elucidated that IL-22 is involved in tumorigenesis and tumor progression in liver, pancreatic and colon cancers [21–23]. In addition, it has been shown that IL-22 increased glucose uptake in brown adipose tissue [24, 25]. However, the effect of IL-22 on aerobic glycolysis in cancer cells has not been investigated until date.

In the present study, we sought to determine whether and how aberrant expression of IL-22 could potentially promoted aerobic glycolysis. We demonstrated that IL-22 enhances aerobic glycolysis via targeting hexokinase 2 (HK2) in colon cancer cells. We further showed that up-regulated HK2 could partly account for increased proliferation induced by IL-22. Thus, our results establish a previously unappreciated mechanism by which the pro-inflammatory cytokine IL-22 facilitates aerobic glycolysis associated tumor progression.

## RESULTS

### Level of glycolysis and expression of IL-22 in colon cancer tissues

**Figure 1:**
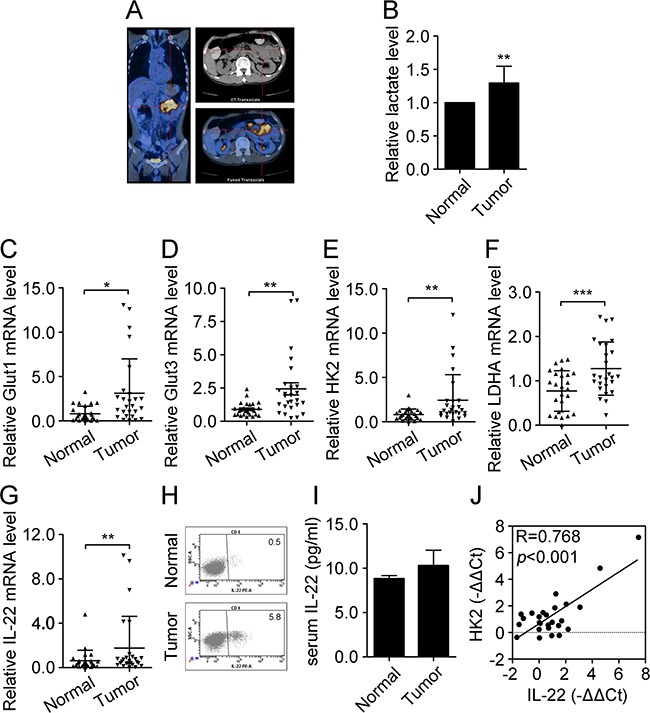
Level of glycolysis and expression of IL-22 increased in colon cancer tissues **(A)** Glucose absorption in colon tumor tissues and adjacent normal tissues were indicated by a PET-CT scan of patient with colon cancer. **(B)** Lactate production was measured in colon tumor and adjacent normal tissues (*n*=10). **(C-F)** Relative mRNA expression of glycolysis-related genes including Glut1 **(C)**, Glut3 **(D)**, HK2 **(E)**, and LDHA **(F)** were determined by RT-PCR in colon tumor tissues and adjacent normal tissues (*n*=26). Actin was used as an internal reference. **(G)** Relative mRNA expression of IL-22 was determined by RT-PCR in colon tumor tissues and adjacent normal tissues (n=26). **(H)** Flow cytometry analysis of IL-22 protein expression was performed in colon tumor tissues. **(I)** Serum IL-22 was measured by ELISA in normal donors (n=7) and patients with colon cancer (n=7) with borderline significance (p=0.058). **(J)** Correlation analysis between IL-22 mRNA expression and HK2 mRNA expression in colon cancer tissues (*n*=26). *p< 0.05, **p< 0.01.

A PET-CT scan of a patient with colon cancer showed elevated glucose absorption in the colon tumor tissues (Figure 1A). As expected, the levels of lactate were found to be higher in tumor tissues than in the adjacent normal tissues (Figure 1B). In an attempt to understand this at a gene-expression level, 26 colon cancer tissues samples, paired with their adjacent normal colon tissues, were obtained and the mRNA levels of glycolytic genes were detected in each by qRT-PCR. As shown in Figure 1C-1F, the mRNA levels of Glut1, Glut3, HK2, and lactate dehydrogenase A (LDHA) were dramatically higher in tumor tissues than in the adjacent normal tissues. Notably, the mRNA level of IL-22 was also significantly up-regulated in tumor tissues, and this was confirmed by the results of flow cytometry and ELISA (Figure 1G-1I). Correlation analysis revealed that IL-22 expression was positively correlated with the expression of HK2 (R=0.768, p<0.001) (Figure 1J). Thus, these data demonstrated that IL-22 and glycolysis-related genes are aberrantly expressed in colon tumor tissues, indicating that they may play important roles in the development of human colon cancer.

### IL-22 enhances aerobic glycolysis in colon cancer cells via targeting HK2

**Figure 2:**
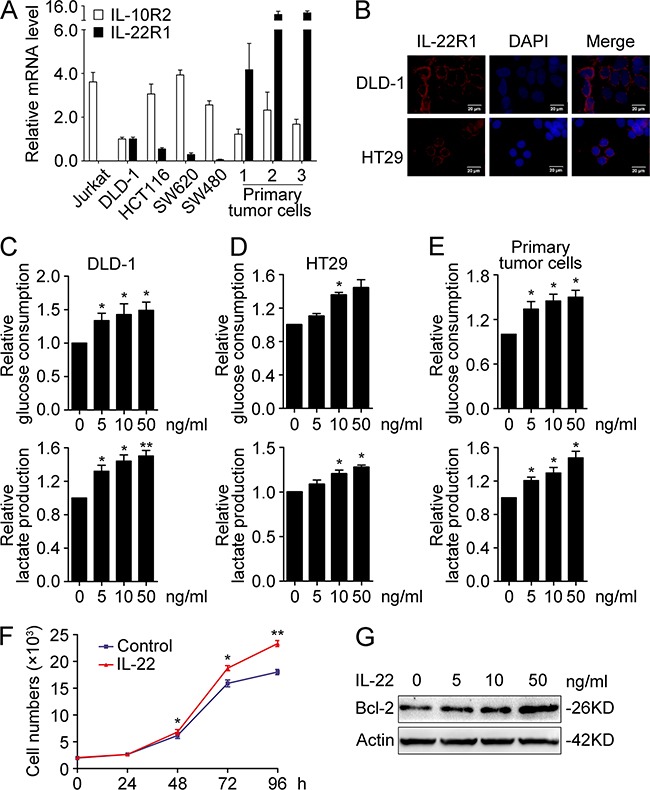
IL-22 promoted aerobic glycolysis associated with cellular proliferation in colon cancer cells **(A)** Relative mRNA expression of IL-22R1 and IL-10R2 were determined in colon cancer cell lines (DLD-1, HCT116, HT29, SW480, SW620, and three primary tumor cells) and the lymphoma cell line Jurkat by RT-PCR. The mRNA expression in DLD-1 cells was used for the normal control. Actin was used as an internal reference. **(B)** The cellular localization of the IL-22R1 protein was determined in DLD-1 and HT29 cells by immunofluorescent assay. **(C-E)** Colon cancer cells were treated with either recombinant IL-22 (10ng/ml) or PBS for 24h. The culture supernatants were harvested and measured for D-glucose consumption (upper) and L-lactate production (down) in DLD-1 **(C)**, HT29 **(D)** and primary tumor cells **(E)**. **(F-G)** IL-22 enhanced cellular proliferation **(F)** and Bcl-2 protein expression **(G)** in DLD-1 cells. One representive result of three independent experiments was presented. *p< 0.05, **p< 0.01.

**Figure 3:**
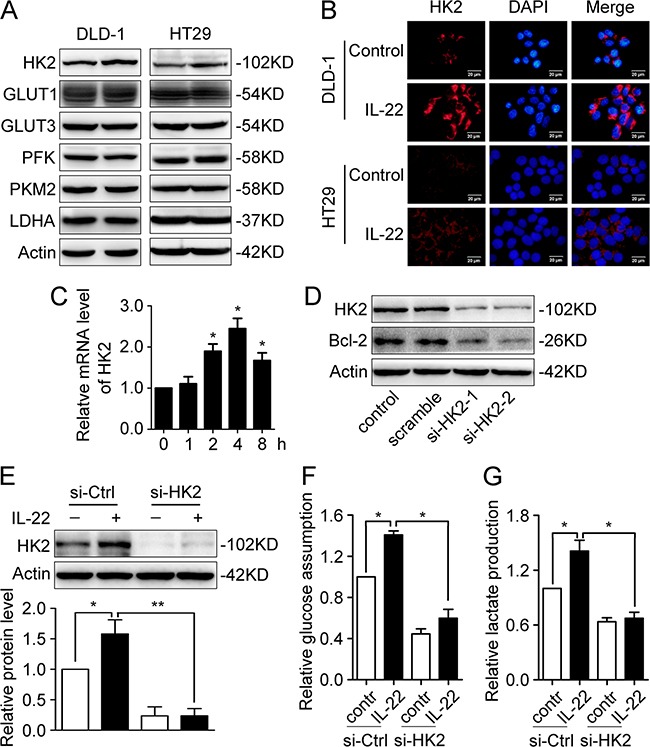
IL-22 enhances aerobic glycolysis by up-regulating HK2 **(A)** Glycolysis-related proteins (HK2, Glut1, Glut3, PFK, PKM2 and LDHA) were determined by western blot in DLD-1 and HT29 cells stimulated with either IL-22 (10ng/ml) or PBS for 24h. Actin served as loading control. **(B)** The cellular localization of the elevated HK2 protein was determined by immunofluorescent assay in DLD-1 and HT29 cells stimulated with either IL-22 (10ng/ml) or PBS for 24h. **(C)** HK2 mRNA level was determined by RT-PCR in DLD-1cells stimulated with either IL-22 (10ng/ml) or PBS for 24h. Actin was used as an internal reference. **(D)** HK2 was knocked down by siRNA in DLD-1 cells. **(E)** The elevated HK2 protein level induced by IL-22 was significantly decreased in si-HK2 group compared with control group. **(F-G)** Knockdown of HK2 attenuated the effect of IL-22 on glucose consumption **(F)** and lactate production **(G)** in DLD-1 cells. One representive result of three independent experiments was presented. *p< 0.05, **p< 0.01.

We speculated that aberrant expression of IL-22 may be involved in glucose metabolism reprogramming in colon cancer cells. To test our hypothesis, experiments were conducted in several colon cancer cell lines. qRT-PCR and immunofluorescence demonstrated IL-22R1 is expressed in these cell lines (Figure 2A and 2B). Glucose consumption and lactate production were measured in culture supernatants of DLD-1, HT29 and primary tumor cell stimulated with either recombinant IL-22 or PBS. As showed in Figure 2C-2E, in colon cancer cells, IL-22 significantly promoted both glucose consumption and lactate production in a dose-dependence manner. Next, we analysed the expression of glycolytic enzymes in DLD-1 and HT29 cells. Our results showed that IL-22 significantly promoted both the mRNA and protein expression of HK2 (Figure 3A-3C), indicating that HK2 is a target gene of IL-22. Moreover, immunofluorescence revealed that the elevated HK2 was mainly localized in the cytoplasm of cancer cells, which is the primary worksites of HK2 (Figure 3B). This data was in accordance with the result of the correlation analysis of genes expression in clinical samples (Figure 1J). Knockdown of HK2 by siRNA attenuated the promotion of IL-22 on glucose consumption and lactate production (Figure 3D-3G), indicating that HK2 is a functional downstream target of IL-22 in facilitating aerobic glycolysis of cancer cells.

### c-Myc mediates IL-22-induced up-regulation of HK2

**Figure 4:**
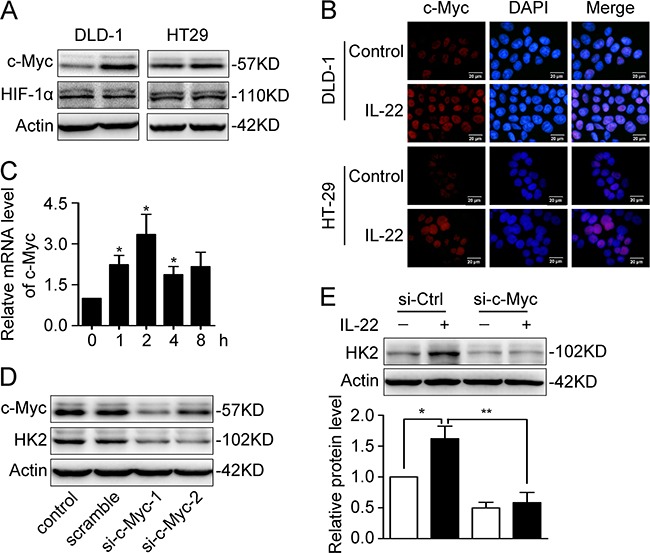
c-Myc was responsible for IL22-induced upregulation of HK2 **(A)** c-Myc and HIF-1α protein level was determined by Western Blot in DLD-1 and HT29 cells stimulated with either IL-22 or PBS. Actin served as loading control. **(B)** The cellular localization of the elevated c-Myc protein was determined by immunofluorescent assay in DLD-1 and HT29 cells stimulated witheither IL-22 (10ng/ml) or PBS for 4h. **(C)** c-Myc mRNA level was determined by RT-PCR in DLD-1cells stimulated with either IL-22 (10ng/ml) or PBS. Actin was used as an internal reference. **(D)** c-Myc was knocked down by siRNA in DLD-1 cells. **(E)** The elevated HK2 protein level induced by IL-22 was significantly decreased in si-c-Myc group compared with control group. One representive result of three independent experiments was presented. *p< 0.05, **p< 0.01.

To further understand at the transcriptional level, the expression levels of c-Myc and HIF-1, two master transcriptional regulators of glycolysis-related genes, were determined. The results showed that c-Myc was up-regulated at both mRNA and protein levels, but that HIF-1 was not affected (Figure 4A-4C). Immunofluorescence analysis showed that up-regulated c-Myc was mainly localised to the nuclei in colon cancer cells (Figure 4B), which facilitated it to exert transcription regulation functions. Based on these data, we surmised that c-Myc may be mainly responsible for the IL-22-induced up-regulation of HK2. To test our assumption, we employed siRNA to decrease c-Myc protein expression (Figure 4D). We found that the up-regulation of HK2 induced by IL-22 was blocked when c-Myc was knocked down in DLD-1 cells (Figure 4E).

### Activated STAT3 is involved in IL-22-enhanced aerobic glycolysis

**Figure 5:**
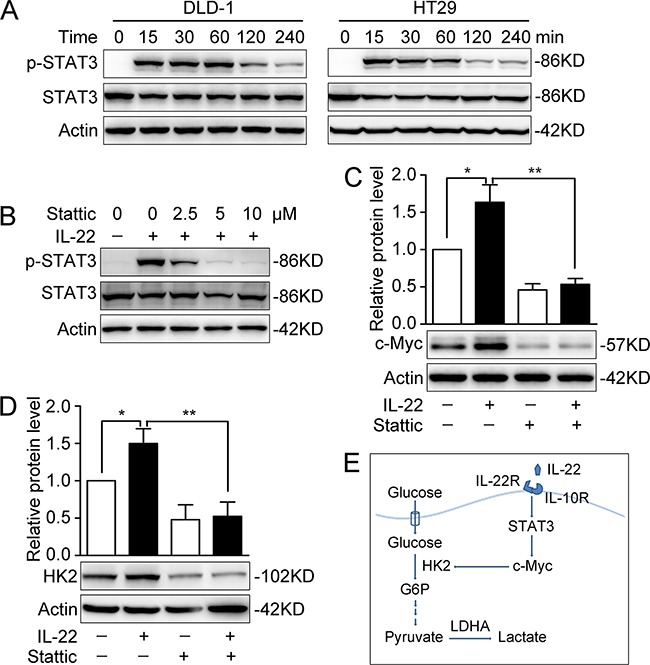
Activated STAT3 was involved in promoting aerobic glycolysis by IL-22 **(A)** STAT3 and P-STAT3 protein levels were determined by Western Blot in DLD-1 and HT29 cells stimulated with either IL-22 (10ng/ml) or PBS for a range of times. Actin served as loading control. **(B)** The protein expression of P-STAT3 was significantly decreased by a specific small molecular inhibitor (stattic). **(C-D)** The elevated c-Myc **(C)** and HK2 **(D)** protein levels induced by IL-22 were significantly decreased in stattic group (pre-treatment with 10 μM stattic for 30min) compared with control group. One representive result of three independent experiments was presented. *p< 0.05, **p< 0.01. **(E)** Summary of this study: results in this study demonstrated that IL-22 regulates aerobic glycolysis through a STAT3/c-Myc/HK2 signaling pathway in colon cancer cells.

To further explore the intracellular signal transduction induced by IL-22, we determined the phosphorylation of STAT3 in DLD-1 and HT29 cells. After treatment with IL-22 for a range of times, STAT3 was found to be phosphorylated significantly, with not changes in total STAT3 (Figure 5A). The level of STAT3 phosphorylation peaked half an hour to an hour following IL-22 treatment. Pre-treatment with a specific small molecular inhibitor (stattic) for 30min significantly inhibited the IL-22-induced phosphorylation of STAT3 in a dose-dependent manner (Figure 5B). Furthermore, the up-regulation of c-Myc and HK2 induced by IL-22 was eliminated by stattic in DLD-1 (Figure 5C and 5D).

### HK-2 partly accounts for IL22-mediated cell proliferation in colon cancer cells

**Figure 6:**
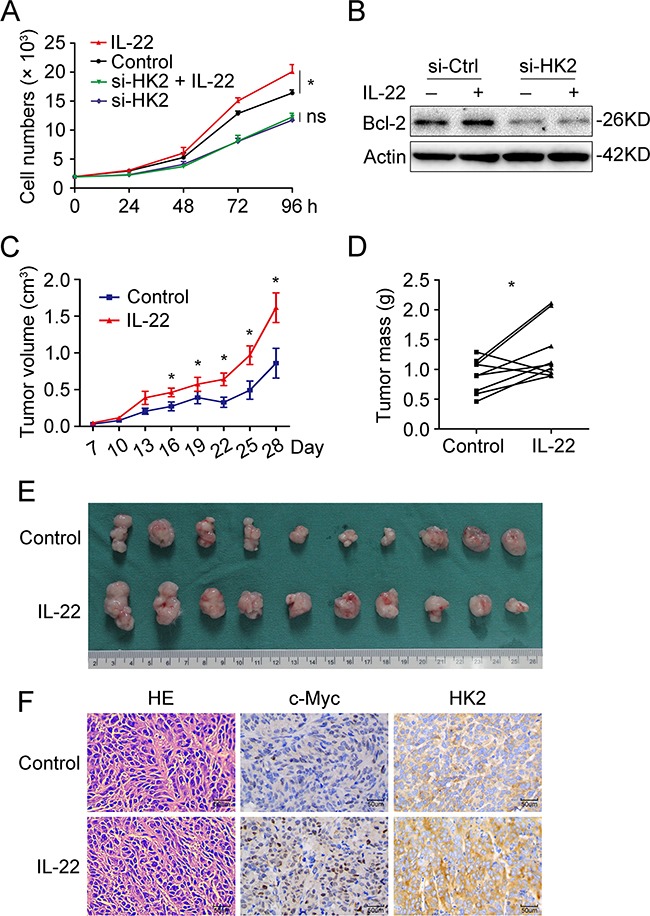
HK-2 partly accounted for IL22-mediated cell proliferation in colon cancer cells **(A)** Cell growth analysis revealed that knockdown of HK2 attenuated the promoting effect of IL-22 on colon cancer cell proliferation. **(B)** The upregulation of Bcl-2 by IL-22 was decreased in si-HK2 group compared with control group. **(C)** Tumor volume was measured every three days from the seventh day after injection. **(D)** Mice were sacrificed after 4 weeks and tumor weights were measured. Paired samples were connected with line. **(E)** Tumor images were displayed. **(F)** The histopathology of tumor tissue was observed with HE-staining and the expression of c-Myc and HK2 in tumor tissues was measured by immunohistochemistry. *p< 0.05.

Since our results had clearly demonstrated that IL-22 enhanced aerobic glycolysis via targeting HK2 in colon cancer cells, we further explored whether HK2 accounts for IL22-mediated cancer cell proliferation. Cell growth assay revealed that knockdown of HK2 attenuated the promoting effect of IL-22 on colon cancer cell proliferation (Figures 6A and 6B). To assess the effects of IL-22 on aerobic glycolysis and tumorigenesis *in vivo*, xenograft experiments were conducted in NOD/SCID mice. IL-22-stimulated DLD-1 cells and paralleled cells were injected subcutaneously into these mice and the resulting tumor size and mass were measured. IL-22 significantly increased both tumor size and mass compared to the control (Figures 6C-6E). Immunohistochemistry of tumor tissues revealed that tumors administrated with IL-22 had higher p-STAT3, c-Myc, and HK2 expression than that in control group (Figure 6F).

## DISCUSSION

The importance of inflammation in development of cancer has been well appreciated, so is the effect of aerobic glycolysis on survival and proliferation of cancer cells. However, the relationship between the two remains largely elusive. In this study, we observed that, in human colon cancer tissues, the aerobic glycolysis was enhanced along with elevated expression of glycolysis-related genes, including HK2. Importantly, we found a relationship between inflammation and aerobic glycolysis. Pro-inflammatory cytokine IL-22 was found to enhance aerobic glycolysis through a STAT3/c-Myc/HK2 signaling pathway in colon cancer cells. In addition, HK-2 was partly responsible for IL22-mediated cell proliferation in DLD-1 cells. Therefore, for the first time, our study has identified IL-22 as an important regulator of metabolic reprogramming in colon cancer cells.

The overexpression of HK-2 has been described in a wide variety of malignant tumors, indicating that HK-2 plays an important role in the development of cancer. In fact, HK2 has been shown to be closely related to the reprogramming of glucose metabolism in variety of cancers [26–28], allowing cancer cells to proliferate and survive though maintenance of biosynthesis and redox homeostasis [4, 5]. Also in the present study, our result showed that HK2 overexpressed in colon cancer tissues and knockdown of HK2 attenuated the stimulatory effect of IL-22 on glucose utilization and lactate production in DLD-1 cells.

HIF-1α and c-Myc are two master transcriptional regulators of glycolysis-related genes. IL-22 selectively up-regulated the expression of c-Myc but not HIF-1α, as previously reported [29]. Ectopic expression of the oncogene c-Myc has been showed in primary colon cancer, and in fact c-Myc overexpression is observed in 60% of patients with colon cancer [30]. In fact, many glycolytic genes targeted by c-Myc, such as enolase 1 (ENO1), HK2, and LDHA, contain conserved canonical E-box binding sites with a high affinity for c-Myc in their proximal promoters [31]. Hence, the transcriptional factor c-Myc may directly bind to transcriptional promoter region of HK2, rendering a metabolic shift toward glycolysis in colon cancer cells. Certainly, further investigations are required to confirm this conclusion.

Notably, our results indicated that IL-22 increased the expression of c-Myc at the mRNA and protein level by activating STAT3. As a member of the IL–10 cytokine family, IL-22 is a potent activator of STAT3, having an important role in tumorigenesis and tumor development [21, 22, 29]. Other than its involvement in regulating cell proliferation, suppressing apoptosis and immune suppression, STAT3 also controls aerobic glycolysis. In ovarian cancer cells, STAT3 overexpression enhanced glucose consumption and lactate production [32]. Furthermore, it has been reported that the transcription factor STAT3 can bind to a region overlapping the E2F site in the c-Myc promoter and thereby activates transcription of c-Myc [33].

Overexpression of Bcl-2 family proteins enhances the survival of cancer cells by inhibiting apoptosis [34, 35]. Numerous studies have shown IL-22 promotes tumor progression through up-regulating Bcl-2 protein expression [21, 22, 36]. In the study, the protein level of Bcl-2 was observed to increase, in accordance with the results of cell proliferative assay. Here, Bcl-2 was just regarded as an indicator of cell survival, which partly accounts for IL-22-induced tumor growth. It has been demonstrated that cell survival-associated Bcl-XL and Bcl-2, proliferation-associated CyclinD1, and VEGF are involved in the promotion effect of IL-22 on tumor [21, 22].

In summary, our data has revealed that pro-inflammatory cytokine IL-22 enhances aerobic glycolysis through a STAT3/c-Myc/HK2 signaling pathway in colon cancer cells. This finding provides a new perspective from which to view the function of IL-22 as a link between inflammation and the Warburg effect. Further, HK2 may be a potential target for therapy in colon cancer with aberrant expression of IL-22.

## MATERIALS AND METHODS

### Clinical samples

33 surgical specimens or peripheral blood specimens were derived from cases of CRC diagnosed and treated in Union Hospital (Wuhan, China) from March 2016 to December 2016. Buffy coats from 7 normal donors were also obtained from the Union Hospital. All samples were obtained and used in presence of the patients’ written informed consent. This study was approved by the Ethics Committee of Wuhan Union Hospital.

### Cell culture

Three primary colon cancer cell lines (1, 2, and 3) were generous gifts from Prof. Weiping Zou (University of Michigan, USA). Colon cancer cell lines DLD-1, HT29, SW480, SW620, HCT116 and the lymphoma cell line Jurkat were obtained from the American Type Culture Collection (ATCC). DLD-1, SW480, SW620, Jurkat and primary colon cancer cell lines were cultured in RPMI-1640 medium (Gibco) containing 10% foetal bovine serum (FBS) (Science cell) and 1% penicillin/streptomycin. HT29 and HCT116 were cultures in McCoy's 5A medium (Gibco) with 10% FBS and 1% penicillin/streptomycin. Cells were maintained in a humidified incubator with 5% CO_2_ atmosphere at 37°C.

### Measurement of glucose uptake and lactate production

Glucose (HK) assay reagent kit (GAHK20, Sigma) and lactate assay kit (K627-100, BioVision) were used to measure the concentration of D-glucose and lactate in cell culture supernatants and sample tissues according to the manufacturer's protocols. Values were normalized to cell number or tissue mass, as appropriate.

### Enzyme linked immunosorbent assay (ELISA)

Serum IL-22 was quantified using an ELISA kit purchased from eBioscience, Inc. (USA) according to the manufacturer's instruction. Briefly, all serum samples were measured in duplicate and the average was adopted to calculate the concentration of IL-22 upon comparison to IL-22 standards provided in the kit.

### Quantitative real-time polymerase chain reaction (qRT-PCR)

Total RNA was extracted from colon tumor tissues, adjacent non-tumorous tissues, and colon cancer cell lines, using RNAiso Plus reagent (Takara) and reverse transcribed to cDNA with PrimeScript™ RT Master Mix Kit (Takara) according to the manufacturer's instructions. qRT-PCR was performed according to the SYBR green method on a StepOnePlus Real-Time PCR System (Applied Biosystems). The mRNA expression of interested genes was normalized to the expression of β-actin. The mRNA relative expression was calculated as 2^−ΔΔ Ct^. All primer sequences used are listed in Table 1.

**Table 1:** Primer sequences used for amplification in this study

Gene	Primer	Sequence	Size
*Glut1*	Forward	TTCACTGTCGTGTCGCTGTTTG	522bp
	Reverse	TCACACTTGGGAATCAGCCCC	
*Glut3*	Forward	TCCCCTCCGCTGCTCACTATTT	190bp
	Reverse	ATCTCCATGACGCCGTCCTTTC	
*HK2*	Forward	AAGATGCTGCCCACCTACG	152bp
	Reverse	TCGCTTCCCATTCCTCACA	
*LDHA*	Forward	TGGCAGATGAACTTGCTCTTGT	184bp
	Reverse	CTTTCTCCCTCTTGCTGACG	
*IL-22*	Forward	GCAGGCTTGACAAGTCCAACT	70bp
	Reverse	GCCTCCTTAGCCAGCATGAA	
*IL-10R2*	Forward	CATTGGGAATGGTACCAC	292bp
	Reverse	CCAATAATGGTGTCATCCAC	
*IL-22R1*	Forward	GTATAAGACGTACGGAGA	373bp
	Reverse	TCCAAGGTGCATTTGGTA	
*C-Myc*	Forward	GGAGGAACAAGAAGATGAGGAAG	132bp
	Reverse	AGGACCAGTGGGCTGTGAGG	
*β-Actin*	Forward	TGGCACCCAGCACAATGAA	186bp
	Reverse	CTAAGTCATAGTCCGCCTAGAAGCA	

### Flow cytometry assay

Colon tumor tissues and adjacent normal tissues were ground and digested to form single cell suspension to detect IL-22 expression by flow cytometry. The following antibodies were used for immunofluorescence staining: PerCP-Cy5.5 conjugated mouse anti-human CD3, APC-Cy7 conjugated mouse anti-human CD4 and PE conjugated mouse anti-human IL-22 (BioLegend).

### Western blotting analysis

Cells were washed twice with cold phosphate buffer saline (PBS) and lysed using cell lysis buffer. Protein concentration was measured with a BCA assay kit (Beyotime). Protein extracts were loaded and separated by 10% SDS-polyacrylamide gel electrophoresis and transferred to polyvinylidenefluoride membranes. Membranes were incubated with specific primary antibodies overnight at 4°C after blocking in 5% non-fat milk as follows: anti-c-Myc, anti-HIF-1α, anti-HK2, anti-Bcl-2, anti-LDHA, anti-phosphorylated STAT3 (Tyr705) and anti-STAT3 (1:1000, CST); anti-Glut1, anti-Glut3, anti-PFK, and anti-PKM2 (1:1000, Abcam); anti-β-actin (1:4000, Sigma). The membranes were incubated with horseradish peroxidase conjugated secondary antibody for 1 h at room temperature, and visualized with enhanced chemiluminescence. The aimed protein levels were normalized to the level of β-actin.

### Immunofluorescence

DLD-1 and HT29 cells were cultured on coverslips in 6-well plates. Cells were incubated with specific primary antibodies overnight at 4°C after being washed, fixed and punched. The following day, the cells were incubated with Cy3 conjugated goat Anti-rabbit IgG for 30 min at room temperature after being washed three times. Cell nuclei were stained with 4, 6-diamidino-2-phenylindole (DAPI) for 5 min. After being washed with PBS three times, the cells were imaged under a fluorescent microscopy equipped with a digital camera.

### siRNA transfection

siRNA oligonucleotides targeting c-Myc and HK2 were purchased from Ruibo Company (Shanghai, China). The targeted sequences of siRNA are as follows: si-c-Myc-1: 5′-GGACTATCCTGCTGCCAAG-3′; si-c-Myc-2: 5′-GGTCAGAGTCTGGATCA CC-3′. si-HK2-1: 5′-GACACAGTCGGAACTATGA-3′; si-HK2-2: 5′-GTGGACAGGATACGAGAAA-3′. DLD-1 cells were transfected with 50 nM siRNA, using Lipofectamine® RNAiMAX Transfection Reagent (Invitrogen) according to the manufacturer's instruction. Twenty-four hours after transfection, cells were trypsinized and re-seeded for follow-up testing.

### Cell growth assay

Cell Counting Kit-8 (CCK-8) was used to determine cell numbers according to the manufacturer's instructions. Briefly, 2 × 10^3^ cells were seeded into 96-well plates containing 100 ul complete medium and allowed to attach overnight. 100 ul complete medium with or without human recombinant IL-22 was then added to each well. At the indicated time, 20 ul CCK-8 was added to each well and incubated at 37°C. After an hour, the absorbance was measured at 450 nm wavelength using a plate reader.

### Animal studies

All animal studies were conducted with approval from the Animal Research Ethics Committee of the Huazhong University of Science and Technology of China. For xenograft experiments, DLD-1 cells were injected subcutaneously into the flanks of non-obese diabetic/severe combined immunodeficiency (NOD/SCID) mice which were randomly assigned to the indicated groups. A week after injection, the tumor size was determined every three days and calculated with the equation, volume = length × width^2^/2. At the end of the experiment, tumors were extracted and tumor mass were measured.

### Immunohistochemistry

Tumor tissues extracted from mice were fixed with paraformaldehyde for HE and immunohistochemical staining. Briefly, after being de-paraffinized and rehydrated, antigen retrieval and neutralization of endogenous peroxidase were performed, followed by blocking with5 % bovine serum albumin for 1 h. Slides were incubated with specific primary antibodies overnight at 4°C, followed by incubation with HRP-labelled anti-rabbit secondary antibody for 1 h at room temperature. Visualisation was performed using 3, 30-diaminobenzidine tetra- hydrochloride and counterstained with haematoxylin.

### Statistical analysis

A paired Student's t-test was performed for comparison of the data between indicated groups. Gene expression correlation was calculated by Pearson's chi-squared test. The data were expressed as mean ± standard deviation (s.d). Statistically significant difference was defined as *P*<0.05.
